# Biological characteristics of ATP5A1 and its pathogenic mechanisms in human diseases: advances in clinical translation

**DOI:** 10.3389/fcell.2026.1862366

**Published:** 2026-07-21

**Authors:** Shu-Yin Yuan, Qi Deng, Hong-Juan Gao, Han-Dong Liu, Hu Tang, Qin Wang, Yu-Ming Liu, Qi-Bing Yan

**Affiliations:** 1 Digestive Disease Center, West China Hospital Sichuan University Jintang Hospital (Jintang First People’s Hospital), Chengdu, China; 2 Emergency Department, West China Hospital Sichuan University Jintang Hospital (Jintang First People’s Hospital), Chengdu, China; 3 Department of Laboratory Medicine, West China Hospital Sichuan University Jintang Hospital (Jintang First People’s Hospital), Chengdu, China

**Keywords:** Atp5a1, biomarker, clinical translation, mitochondrial complex V, pathogenic mechanisms

## Abstract

ATP5A1 acts as a crucial gene that encodes the core α subunit of the mitochondrial F1-ATPase complex to regulate cellular oxidative phosphorylation (OXPHOS) and maintain energy homeostasis. Consequently, its dysregulation—whether through aberrant expression, genomic alterations, or aberrant post-translational modifications—is hypothesized to be a key potential molecular event contributing to the pathogenesis of malignant tumors, neurodegenerative lesions, and metabolic dysregulation.This review systematically summarizes the molecular structure, biological functions, and regulatory networks of ATP5A1. A comprehensive overview of its expression patterns and pathogenic mechanisms across various diseases, including lung, colorectal, liver, and gastric cancers, as well as non-neoplastic conditions, is provided. Besides, emphasis is placed on the clinical translational potential of ATP5A1 as a diagnostic biomarker and therapeutic target, with recent advances in this area critically evaluated. Furthermore, current challenges and limitations in ATP5A1-related research are discussed, and future directions are proposed to facilitate mechanistic investigations and clinical applications. Collectively, this review establishes a comprehensive theoretical framework for understanding the role of ATP5A1 in disease pathogenesis and supports its potential utility in precision diagnostics and therapeutics.

## 1 Introduction

The mitochondrial oxidative phosphorylation (OXPHOS) system is fundamental to cellular energy homeostasis (Smeitink et al., 2001; Kühlbrandt, 2015). As the final component of the electron transport chain, F_1_F_0_-ATP synthase (complex V) functions as a molecular motor that couples the transmembrane proton gradient to the synthesis of adenosine triphosphate (ATP), the universal energy currency of the cell. *ATP5A1* encodes the core α subunit of the F_1_ catalytic domain and is indispensable for both the enzymatic activity and structural integrity of ATP synthase. Traditionally, *ATP5A1* has been classified as a constitutive “housekeeping” gene involved in basal metabolic maintenance (Junge and Nelson, 2015). However, accumulating evidence has redefined ATP5A1 as a highly dynamic central metabolic gatekeeper linking mitochondrial energy metabolism to cell-fate determination. Dysregulation of *ATP5A1*, including altered expression, genetic variation, and aberrant post-translational modifications, has emerged as a key pathological feature underlying the development of complex systemic diseases (Singh, 2023). In malignant tumors, *ATP5A1* exhibits a context-dependent dual role, functioning as either a tumor suppressor or promoter depending on the metabolic landscape. In contrast, in cardiovascular and neurodegenerative disorders, the structural compromise of ATP synthase and the resulting bioenergetic failure are closely associated with excessive oxidative stress and progressive tissue damage. These findings collectively establish *ATP5A1* as central to mitochondrial bioenergetics and disease pathophysiology.

Despite the rapid expansion of molecular studies on ATP5A1, a comprehensive cross-system integration of its biological functions and disease relevance remains lacking. To address this gap, the present review systematically summarizes the molecular structure, core functions, and regulatory networks of *ATP5A1*, and provides an integrated overview of its pathogenic roles across a wide spectrum of diseases, including malignant tumors and non-neoplastic disorders affecting the cardiovascular, neuropsychiatric, metabolic, and reproductive systems. Emphasis is placed on recent advances in clinical translation, highlighting its potential as a liquid biopsy biomarker and a multidimensional therapeutic target. In addition, current research limitations are critically evaluated, and future interdisciplinary directions are proposed to advance mechanistic insights and precision medicine applications. Collectively, this review aims to establish a comprehensive theoretical framework for understanding ATP5A1 and to support its further development in disease diagnosis and targeted intervention.

## 2 Structure and core functions of ATP5A1

### 2.1 Structural characteristics of ATP5A1

The *ATP5A1* gene is located on human chromosome 18q21 and encodes the core α subunit of the F_1_ catalytic domain within the mitochondrial ATP synthase (complex V) (Jonckheere et al., 2013). The structural integrity and spatial conformation of the encoded protein are fundamental to maintaining cellular bioenergetic homeostasis.

#### 2.1.1 Spatial conformation and structural stability

**FIGURE 1:**
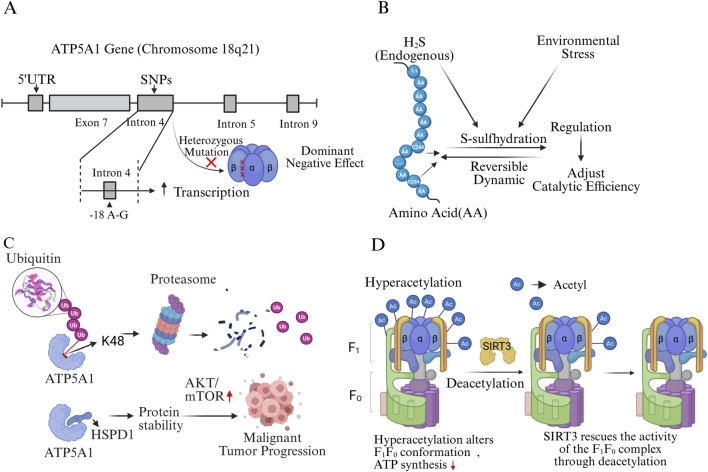
Key regulatory mechanisms of ATP5A1. **(A)** Genetic and transcriptional regulation: Polymorphisms in the ATP5A1 gene, such as the intron 4–18 A→G variant, enhance transcriptional activity, whereas specific heterozygous mutations disrupt complex V assembly through a dominant-negative effect. **(B)** S-sulfhydration modification: In response to environmental stress, endogenous H_2_S induces reversible S-sulfhydration at C244 and C294 residues, thereby dynamically regulating catalytic efficiency. **(C)** Ubiquitination and tumor pathology: The molecular chaperone HSPD1 masks the K48 ubiquitination site, allowing ATP5A1 to evade proteasomal degradation. This aberrant stabilization activates the AKT/mTOR signaling pathway, thereby driving malignant tumor progression. **(D)** Dynamic acetylation: Hyperacetylation alters the catalytic conformation of F_1_F_0_-ATP synthase and reduces ATP synthesis efficiency; in contrast, the deacetylase SIRT3 precisely removes acetyl groups from core sites, thereby rescuing and restoring complex V activity.

The ATP5α subunit alternates with β subunits to form a heterohexameric (α_3_β_3_) assembly, which constitutes the catalytic core of complex V. Current evidence indicates that heterozygous mutations within the coding region, including specific amino acid substitutions, can disrupt the precise assembly of complex V through dominant-negative mechanisms. This disruption impairs the interaction between α and β subunits, ultimately leading to impaired OXPHOS function (Walker, 2013) (Figure 1A).

#### 2.1.2 Genetic polymorphisms and transcriptional regulatory networks

*ATP5A1* exhibits a high degree of genetic polymorphism, with key single-nucleotide polymorphisms (SNPs) distributed across the 5′untranslated region (UTR), exon 7, and multiple intronic regions (introns 4, 5, and 9). Notably, a specific variant located at position −18 in intron 4 (A→G) has been reported to exert a significant transcriptional enhancement effect (Figure 1A). Furthermore, the expression of ATP5A1 is closely associated with the host genomic context, particularly with mutations in TP53. The interplay between ATP5A1 expression and TP53 mutation status is proposed as an important molecular underpinning for the progression of malignant phenotypes in colorectal cancer (Seth et al., 2009).

#### 2.1.3 Key modification sites

The primary structure of ATP5A1 harbors a sophisticated post-translational modification network that governs its enzymatic activity and protein stability. On one hand, specific cysteine residues (e.g., C244 and C294) function as redox sensors and can undergo S-sulfhydration mediated by endogenous H_2_S. This reversible modification dynamically regulates the catalytic efficiency of ATP synthase and represents a critical mechanism by which mitochondrial energy metabolism adapts to environmental stress (Módis et al., 2016) (Figure 1B). On the other hand, lysine residue K48 serves as a central site controlling ubiquitin-mediated degradation. In pathological models such as osteosarcoma, the molecular chaperone HSPD1 physically binds to and masks this K48 site, thereby evading ATP5A1 degradation via the proteasomal pathway. Such pathological stabilization leads to sustained and excessive activation of the AKT/mTOR signaling pathway, ultimately contributing to the molecular framework that promotes malignant tumor progression (Zhang et al., 2024) (Figure 1C).

Furthermore, the catalytic activity of ATP5A1 is tightly regulated by highly dynamic acetylation modifications. Studies have demonstrated that hyperacetylation of ATP5A1 under pathological conditions significantly alters the catalytic conformation of F_1_F_0_-ATP synthase, thereby severely impairing the efficiency of mitochondrial ATP synthesis. In contrast, under physiological or stress-adaptive conditions, the primary mitochondrial deacetylase SIRT3 selectively recognizes and removes acetyl groups from key residues on ATP5A1, thereby rescuing and restoring complex V activity (Wu et al., 2013; Vassilopoulos et al., 2014) (Figure 1D).

### 2.2 Core functions of ATP5A1

As an integral component of mitochondrial complex V in eukaryotic cells, ATP5A1 serves as an allosteric hub for energy conversion and a key executor of mitochondrial ultrastructural homeostasis and cell fate determination (Caron and Bertolin, 2024). Its core functions can be summarized into the following three dimensions.

#### 2.2.1 Allosteric anchor for mechanochemical coupling

**FIGURE 2:**
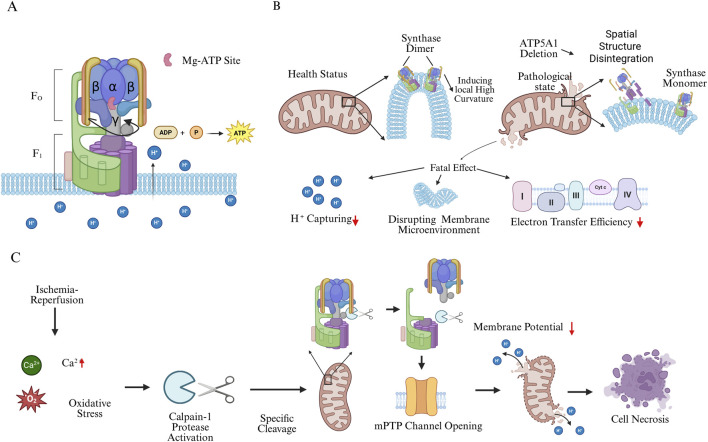
Core functional mechanisms of ATP5A1. **(A)** Mechanochemical coupling: ATP5A1 serves as an allosteric anchor that confers structural rigidity to the F_1_ domain, thereby ensuring efficient rotational coupling and preventing futile slippage of the molecular motor. **(B)** Cristae morphology remodeling: As a structural scaffold, ATP5A1 stabilizes complex V dimers, induces inner membrane curvature, and maintains cristae architecture; its dissociation leads to cristae collapse and respiratory chain dysfunction. **(C)** Structural decoupling mediates mPTP opening: Calpain-1 specifically cleaves ATP5A1, resulting in mechanical uncoupling between the F_1_ catalytic head and the F_0_ rotor. Structural damage to ATP5A1 triggers the formation of the high-conductance mPTP, leading to loss of mitochondrial membrane potential, matrix swelling, and tissue necrosis.

Within the F_1_ catalytic head of mitochondrial complex V, three α subunits encoded by ATP5A1 alternate with three β subunits to form an α_3_β_3_ hexameric catalytic ring. ATP5A1 binds non-catalytic Mg-ATP and functions as a critical structural anchoring element. As proton flux drives the rotation of the central γ subunit, the structural rigidity of ATP5A1 imposes precise conformational cycling on the adjacent β subunits, thereby efficiently driving the condensation of ADP and inorganic phosphate. Thus, ATP5A1 serves as a crucial component that prevents rotational slippage and ensures the conversion of mechanical energy into chemical energy (Abrahams et al., 1994; Noji et al., 1997; Capaldi and Aggeler, 2002) (Figure 2A).

#### 2.2.2 Physical remodeling of mitochondrial cristae morphology

On the inner mitochondrial membrane of healthy cells, ATP synthase assembles into dimers or higher-order oligomers that are specifically enriched at the tips of mitochondrial cristae. This supramolecular organization induces local high membrane curvature and provides the physical basis for maintaining the characteristic tubular or lamellar architecture of cristae (Strauss et al., 2008; Blum et al., 2019). As a structural cornerstone of the F_1_ domain, ATP5A1 is essential for the stability of the V complex dimer. Loss of ATP5A1 expression or aberrant protein folding directly leads to the loosening and disassembly of complex V dimers, culminating in the complete collapse of the cristae architecture. Such ultrastructural disruption not only impairs local proton-trapping capacity but also triggers a deleterious cascade: it disrupts the membrane microenvironment, compromises the assembly stability of respiratory chain supercomplexes, and reduces electron-transfer efficiency, thereby markedly exacerbating cellular energy deficiency (Cogliati et al., 2013) (Figure 2B).

#### 2.2.3 Mediation of mPTP opening

From a contemporary pathophysiological perspective, F_1_F_0_-ATP synthase has been identified as the core molecular entity of the mitochondrial permeability transition pore (mPTP) (Bernardi et al., 2015; Giorgio et al., 2013). Under conditions of calcium overload and intense oxidative stress, such as those induced by ischemia-reperfusion injury, ATP5A1 is highly susceptible to specific pathological modifications, including proteolytic cleavage by aberrantly activated calpain-1 (Liu et al., 2022; Zhang et al., 2021). This physical disruption of ATP5A1 leads to mechanical uncoupling between the F_1_ catalytic head and the F_0_ transmembrane channel, thereby strongly facilitating the irreversible opening of the high-conductance mPTP. Consequently, mitochondrial membrane potential collapses, matrix swelling occurs, and pro-apoptotic factors are released, ultimately driving tissue necrosis and inflammatory remodeling (Ni et al., 2016; Halestrap and Richardson, 2015; Baines et al., 2005) (Figure 2C).

## 3 Disease-associated expression and pathogenic mechanisms of ATP5A1

### 3.1 Functional roles and mechanisms of ATP5A1 in tumors

The expression profile of *ATP5A1* in malignant tumors exhibits pronounced tissue specificity and functional duality. In certain contexts, ATP5A1 is downregulated in colorectal cancer, clear cell renal cell carcinoma, and hepatocellular carcinoma, where reduced expression levels are frequently associated with more advanced clinicopathological features and unfavorable prognosis. Conversely, in tumors with high metabolic demands, such as lung cancer, gastric cancer, glioblastoma, and triple-negative breast cancer, ATP5A1 is pathologically overexpressed and actively participates in tumor cell proliferation and invasion. The expression patterns, core functions, and molecular mechanisms of ATP5A1 in selected malignancies are summarized in Table 1.

**TABLE 1:** Expression patterns, core functions, and molecular mechanisms of ATP5A1 in selected malignancies.

Tumor type	Expression status	Core functions and molecular mechanisms	Sample types	Limitations and potential biases	References
Colorectal cancer	Downregulated	Genetically linked with APC; loss of heterozygosity leads to early mitochondrial dysfunction; elevated expression coupled with TP53 mutations drives specific subtypes	Clinical colon adenocarcinoma tissues and adjacent normal tissues; *Apc*Min/+, Mom2R/+ mouse models	Animal models (e.g., *Apc*Min/+ mice) primarily reflect the familial adenomatous polyposis pathway and fail to fully recapitulate the complex mutational evolutionary spectrum of human sporadic colorectal cancer; furthermore, the potential impact of the gut microbiota on mitochondrial metabolism remains unevaluated	Seth et al. (2009), Baran et al. (2007)
Clear cell renal cell carcinoma	Downregulated	Knockdown activates the Wnt/β-catenin signaling pathway, enhancing proliferation and invasion	Clinical ccRCC tissues; human renal cancer cell lines (786-O, CAKI-1, 769-P); HEK293 cells	Mechanistic investigations rely heavily on *in vitro* cell lines, thereby failing to recapitulate the highly characteristic vascularized microenvironment of the tumor and the dynamic regulatory networks of hypoxia-inducible factors (HIFs) within the stroma	Zhou et al. (2025)
Hepatocellular carcinoma	Context-dependent/dual	BRG1 upregulates ATP5A1 to support proliferative energy demands; AKT–ATG4B axis phosphorylation suppresses its activity, inducing ROS accumulation and promoting glycolytic shift	Clinical hepatocellular carcinoma tissuesHuman; HCC cell lines (HuH-7, HepG2)	The HepG2 cell line is inherently derived from hepatoblastoma and may not fully represent the metabolic characteristics of adult primary HCC; furthermore, there is a lack of immunocompetent mouse models to evaluate the interplay between metabolic reprogramming and immune evasion	Hui et al. (2024), Ni et al. (2018)
Lung adenocarcinoma	High (metastatic lesions)	Maintains high ATP production and stable mitochondrial membrane potential, driving invasion, distant colonization, and stress tolerance	Clinical lung adenocarcinoma tissues and metastatic lesions; human A549 cell line	The A549 cell line fails to fully recapitulate the spatiotemporal cascade of distant metastasis; furthermore, clinical samples of metastatic lesions are predominantly acquired retrospectively, rendering them highly susceptible to confounding biases from metabolic remodeling induced by prior chemotherapy or targeted therapies	Pasternack et al. (2023)
Gastric cancer	High	Activates the JNK/JUN signaling pathway, upregulates key glycolytic rate-limiting enzymes (HK2, PFK1, LDHA), remodels the Warburg effect, and provides energy and biosynthetic substrates	Clinical GC tissues; human MGC803 cell line; xenograft tumor nude mouse models	Conventional subcutaneous nude mouse xenograft models lack the gastric-specific orthotopic microenvironment (such as gastric acid-induced low-pH stress and unique stromal interactions); additionally, their immunodeficient state precludes the recapitulation of the immunosuppressive effects exerted by tumor metabolites on the microenvironment	Yang et al. (2024)
Glioblastoma	High (tumor cells and vasculature)	Epigenetic silencing of tumor-suppressive microRNAs (Let-7f, miR-16, miR-100) enhances OXPHOS, supporting hypoxic invasion and promoting microvascular formation	Clinical GBM tumor tissues; GBM-associated microvascular tissues	There is a lack of dynamic *in vivo* animal tracking models capable of faithfully recapitulating the intact blood-brain barrier (BBB) and the complex hypoxic metabolic gradients within the brain	Xu and Li (2016)
Triple-negative breast cancer	High	Maintains extremely high OXPHOS flux, providing robust energy support for invasion and microenvironmental adaptation	Clinical breast cancer tissues; human breast cancer cell lines (MCF-7, SKBR-3, MDA-MB-231, MDA-MB-468, HS578)	Despite the inclusion of multiple established cell lines, conventional *in vitro* culture systems fail to recapitulate the biomechanical stress exerted by the dense breast tumor stroma and the highly heterogeneous tumor-stromal metabolic symbiotic network	Malyarenko et al. (2026)
Osteosarcoma	High	The molecular chaperone HSPD1 masks the K48 ubiquitination site to evade proteasomal degradation, thereby sustaining the activation of the AKT/mTOR signaling pathway and driving malignant tumor progression	Clinical osteosarcoma tissues	Current conventional subcutaneous or orthotopic animal models fail to faithfully recapitulate the highly complex bone matrix microenvironment, as well as the metabolic remodeling crosstalk between osteoclasts/osteoblasts and tumor cells	Zhang et al. (2024)

#### 3.1.1 Colorectal cancer

Clinical multi-omics analyses have demonstrated that ATP5A1 expression is significantly lower in colon adenocarcinoma tissues than in adjacent normal tissues. This downregulation is not only closely associated with advanced tumor stage but also serves as an independent predictor of poor overall survival. Conversely, overexpression of ATP5A1 in cellular and animal models markedly suppresses colorectal cancer growth (Zhang et al., 2022). At the genetic and molecular regulatory level, specific SNPs within the *ATP5A1* gene, such as the intron 4 A→G variant, significantly enhance its transcriptional activity. Moreover, elevated ATP5A1 expression is tightly coupled with TP53 mutations and chromosomal instability, and their synergistic interaction constitutes the molecular basis for malignant phenotypic progression in specific colorectal cancer subtypes (Seth et al., 2009). Due to the close chromosomal linkage between *ATP5A1* and the oncogene *Apc*, loss of heterozygosity during tumor initiation in intestinal epithelial cells simultaneously eliminates the sole functional *ATP5A1* allele. This genetically linked “collateral lethal” effect profoundly impairs mitochondrial function and death of tumor-initiating cells, resulting in an approximately 90% reduction in intestinal polyp burden. These findings provide genetic evidence establishing the indispensable role of ATP5A1 in early tumorigenesis and cell survival (Baran et al., 2007). Based on this metabolic dependency, ATP5A1 has emerged as a promising therapeutic target. Recent studies have demonstrated that engineered mitochondria-targeting peptides can selectively interact with the ATP5A1/SIRT3 complex and disrupt ATP5A1 deacetylation, thereby profoundly impairing mitochondrial homeostasis. Such strategies exhibit potent antitumor activity in both *in vitro* and *in vivo* models of colorectal cancer (Kim et al., 2020).

#### 3.1.2 Clear cell renal cell carcinoma

In clear cell renal cell carcinoma (ccRCC), tumor cells undergo profound metabolic reprogramming characterized by impaired OXPHOS, enhanced glycolysis, and abnormal lipid accumulation. Suppression of ATP5A1 expression represents a central event in this metabolic alteration. Large-scale clinical transcriptomic and histological cohort analyses consistently demonstrate that ATP5A1 expression is significantly downregulated in ccRCC tissues compared with normal kidney tissues. Moreover, reduced ATP5A1 levels are negatively correlated with advanced clinical stage and higher histological grade and serve as an independent biomarker of poor prognosis (Yuan et al., 2018). At the molecular level, ATP5A1 silencing not only reflects mitochondrial bioenergetic defects but also actively drives malignant tumor progression. Functional studies have shown that ATP5A1 knockdown further disrupts intracellular metabolic homeostasis and aberrantly activates the canonical Wnt/β-catenin signaling pathway, thereby significantly enhancing the proliferation, migration, and invasion capacities of ccRCC cells. In contrast, restoration of ATP5A1 expression effectively reverses these malignant phenotypes (Zhou et al., 2025). These findings collectively indicate that ATP5A1 plays a distinct “metabolic tumor suppressor” role in ccRCC. Loss of its expression represents a critical adaptation that enables tumor cells to tolerate hypoxic microenvironments and acquire metabolic flexibility. Pharmacological strategies aimed at restoring *ATP5A1*-mediated mitochondrial homeostasis or inhibiting the downstream Wnt/β-catenin signaling axis may offer promising approaches for the precision treatment of ccRCC.

#### 3.1.3 Hepatocellular carcinoma

In hepatocellular carcinoma (HCC), ATP5A1 functions as a highly dynamic and context-dependent metabolic regulator that governs mitochondrial metabolic reprogramming and the acquisition of malignant phenotypes. On the one hand, aberrant enrichment of ATP5A1 is frequently hijacked by oncogenic signals to meet the energy demands of rapid tumor proliferation and distant metastasis. For example, the chromatin remodeling factor BRG1 activates the TOMM40/ATP5A1 signaling axis, significantly enhancing mitochondrial membrane potential and inhibiting mPTP opening, thereby potently driving HCC cell proliferation and invasion (Hui et al., 2024). On the other hand, targeted inhibition of ATP5A1 catalytic activity represents a key mechanism by which HCC cells actively induce the Warburg effect to acquire metabolic flexibility. Studies have demonstrated that AKT-mediated phosphorylation of ATG4B at Ser34 promotes its translocation to mitochondria, where it specifically binds to and inhibits F_1_F_0_-ATP synthase activity. This suppression of ATP5A1-associated energy production leads to aberrant elevation of mitochondrial ROS, forcing tumor cells to shift toward aerobic glycolysis and thereby enhancing their invasiveness and chemoresistance (Ni et al., 2018). Collectively, these multidimensional mechanisms indicate that ATP5A1 serves as a critical metabolic mediator influencing the fate of HCC cells. Consequently, therapeutic modulation of the ATP5A1-mediated bioenergetic network represents a highly promising translational direction for precision therapy of HCC.

#### 3.1.4 Lung cancer

Clinical proteomic analysis reveals that ATP5A1 exhibits extremely significant high expression in the distant metastatic lesions of lung adenocarcinoma, which suggests that it may be involved in maintaining high levels of ATP supply and a stable mitochondrial membrane potential, thereby pathophysiologically supporting lung cancer cell invasion, distant colonization, and survival tolerance to metabolic stress (Pasternack et al., 2023). At the molecular level, aberrant ATP5A1 enrichment not only establishes an energy-supporting barrier in lung cancer cells but also renders it a potential therapeutic vulnerability for targeted antitumor intervention. For example, the anticancer compound periplocin specifically downregulates ATP5A1 protein abundance in lung cancer cells. This targeted disruption of cellular energy homeostasis rapidly collapses mitochondrial membrane potential and induces lethal ROS accumulation, thereby strongly activating mitochondria-dependent intrinsic apoptotic pathways and suppressing tumor growth (Lu et al., 2014). These findings indicate that targeting ATP5A1-mediated energy metabolism is a promising translational strategy to overcome metabolic adaptation and inhibit malignant progression in lung cancer.

#### 3.1.5 Gastric cancer

In the malignant progression of gastric cancer (GC), ATP5A1 exhibits significant oncogenic potential. Its aberrant high expression is significantly correlated with poor patient prognosis, and it is considered one of the key molecular events that may promote metabolic reprogramming in GC cells. Clinicopathological and survival analyses have demonstrated that ATP5A1 is highly enriched in GC tissues and correlates with advanced TNM stage, higher tumor marker levels, and poorer five-year overall survival, serving as an independent prognostic risk factor. At the molecular level, ATP5A1 not only participates in mitochondrial energy production but also fuels malignant proliferation by remodeling the Warburg effect. Experimental evidence suggests that ATP5A1 overexpression potently activates the downstream JNK/JUN signaling pathway, thereby transcriptionally upregulating key glycolytic rate-limiting enzymes, including hexokinase 2 (HK2), phosphofructokinase 1 (PFK1), and lactate dehydrogenase A (LDHA). This results in significantly increased glucose uptake and lactate production in GC cells (Yang et al., 2024). The ATP5A1–JNK–JUN axis-mediated enhancement of glycolysis provides abundant energy and biosynthetic substrates for rapid tumor expansion. Genetic knockdown of ATP5A1 or pharmacological inhibition of JNK (e.g., SP600125) effectively disrupts this aberrant metabolic oncogenic network and significantly suppresses GC growth *in vitro* and *in vivo*. These findings highlight the central role of ATP5A1 in integrating mitochondrial bioenergetics with cytosolic glycolysis in GC and support its potential as a promising target for precision therapeutic strategies aimed at metabolic vulnerabilities.

#### 3.1.6 Glioblastoma

In glioblastoma (GBM), a highly aggressive malignant brain tumor, ATP5A1 not only serves as a core driver of aberrant tumor energy metabolism but also functions as a critical molecular hub promoting microvascular proliferation. Clinicopathological and multi-omics analyses have revealed that ATP5A1 is markedly overexpressed in both GBM tumor cells and tumor-associated microvascular endothelial cells. This overexpression is not primarily driven by genomic amplification or mutation but rather by aberrant epigenetic silencing of tumor-suppressive microRNAs targeting ATP5A1, including Let-7f, miR-16, and miR-100 (Xu and Li, 2016). At the molecular level, substantial enrichment of ATP5A1 significantly enhances mitochondrial OXPHOS efficiency, thereby meeting the enormous energy demands of sustained invasion and maintenance of tumor stemness under severely hypoxic conditions in the brain microenvironment. In parallel, ATP5A1 promotes the formation of dense microvascular networks that provide nutritional support to the tumor (Xu and Li, 2016). Based on this unique dual metabolic dependency, ATP5A1 has emerged as an attractive dual-target therapeutic candidate in GBM. Recent studies have demonstrated that the mitochondria-targeting peptide P-bi-TAT can specifically and effectively downregulate ATP5A1 and other electron transport chain-related genes in GBM cells. This intervention simultaneously induces energy imbalance in tumor stem cells and disrupts tumor-associated microvasculature, thereby providing a strong translational foundation for improving the prognosis of this refractory central nervous system malignancy (Glinsky et al., 2022).

#### 3.1.7 Triple-negative breast cancer

In breast cancer, particularly the highly aggressive triple-negative breast cancer (TNBC), aberrant enrichment of ATP5A1 and reprogramming of mitochondrial energy metabolism constitute a core dependency for tumor cell survival and malignant proliferation. Studies have demonstrated that TNBC cells maintain extremely high OXPHOS flux by upregulating ATP5A1 and its associated complexes, thereby providing a robust energy foundation for tumor invasion and adaptation to the tumor microenvironment (Malyarenko et al., 2026). Targeted knockdown of ATP5A1 significantly impairs this metabolic adaptability and markedly increases tumor cell sensitivity to chemotherapy (Pateras et al., 2023). At the molecular and therapeutic levels, ATP5A1 has been identified as a direct pharmacological target of novel anti-TNBC agents, including the plant-derived sesquiterpene lactone deoxyelephantopin (DET) and its derivative DETD-35. Molecular docking and proteomic analyses have revealed that DET and DETD-35 specifically bind to the α/β subunit interface of ATP synthase, corresponding to the catalytic core containing ATP5A1. This interaction not only significantly downregulates ATP5A1 protein levels in tumor tissues but also directly inhibits its ATP synthesis activity (Shiau et al., 2026). This targeted disruption of ATP5A1 rapidly induces irreversible mPTP opening, triggers excessive superoxide generation, and causes acute ATP depletion, thereby effectively suppressing TNBC cell proliferation and metastasis through severe mitochondrial bioenergetic collapse. Collectively, these findings underscore the fundamental role of ATP5A1 in metabolic adaptation in breast cancer and provide robust translational evidence for developing mitochondria-targeted therapeutic strategies to disrupt tumor energy supply networks.

#### 3.1.8 A unifying mechanistic framework for the dual role of ATP5A1 in tumors: spatiotemporal trade-offs in microenvironmental adaptation and metabolic evolution

Although ATP5A1 functions as a metabolic tumor suppressor in colorectal cancer and clear cell renal cell carcinoma, while acting as a potent oncogenic driver in gastric cancer, glioblastoma, and triple-negative breast cancer, this context dependency is not a stochastic phenomenon. Based on the aforementioned cross-cancer molecular evidence, we propose a unifying mechanistic framework to elucidate the duality of ATP5A1: the functional transition of ATP5A1 essentially represents a spatiotemporal trade-off executed by tumor cells to achieve maximal metabolic fitness under varying genetic backgrounds, microenvironmental stresses, and stages of progression. This framework comprises the following three core logical tiers:

1. Underlying Determination by Genetic Linkage and Epigenetics (Early Events)

During the early stages of tumorigenesis, the expression pattern of ATP5A1 is frequently dictated by the physical characteristics of the host genome. For instance, in colorectal cancer, due to the tight chromosomal linkage between ATP5A1 and the core oncogene Apc, intestinal epithelial cells “passively” lose the ATP5A1 allele during the loss of heterozygosity that initiates carcinogenesis (Baran et al., 2007). This genetic linkage-mediated loss forces tumor-initiating cells to confront the risk of mitochondrial functional collapse at an extremely early stage. Conversely, in tumors lacking this linkage effect (e.g., glioblastoma), tumor cells “actively” upregulate ATP5A1 through epigenetic reprogramming (such as the silencing of tumor-suppressive miRNAs) to establish a bioenergetic advantage:

2. Microenvironmental Responses Driven by ROS and the Warburg Shift (Compromise in Metabolic Reprogramming)

In solid tumors facing severe microenvironmental hypoxia or intense immune pressure (such as clear cell renal cell carcinoma and hepatocellular carcinoma), the downregulation of ATP5A1 constitutes an active compromise to acquire metabolic flexibility. The suppression or phosphorylation-mediated inactivation of ATP5A1 (e.g., via the AKT-ATG4B axis) creates a terminal bottleneck in oxidative phosphorylation, inducing a moderate release of mitochondrial reactive oxygen species (ROS). This ATP5A1 downregulation-induced metabolic stress not only robustly activates canonical oncogenic pathways (such as Wnt/β-catenin) but also compels a global metabolic shift toward aerobic glycolysis (the Warburg effect). In this context, ATP5A1 functions as a “metabolic tumor suppressor,” and its loss serves as the molecular switch that activates the glycolytic pathway:

3. Absolute Energy Dependency During Invasion and Metastasis (Drivers of Late-Stage Progression)

As tumors progress to stages of rapid proliferation, high invasiveness, or distant metastasis (e.g., metastatic lesions in lung adenocarcinoma and triple-negative breast cancer), the inefficient energy yield of glycolysis alone becomes insufficient to meet the massive bioenergetic and biosynthetic demands of tumor cells. At this stage, tumor clones exhibit a profound dependency on ATP5A1, aberrantly enriching its expression to maintain extremely high oxidative phosphorylation flux and stable mitochondrial membrane potential, thereby supporting microangiogenesis and metastatic colonization. More importantly, during this phase, highly expressed ATP5A1 is no longer mutually exclusive with glycolysis. Instead, it synergistically drives the expression of glycolytic rate-limiting enzymes at the upstream transcriptional level by activating signaling axes such as JNK/JUN (as observed in gastric cancer).

The duality of ATP5A1 can thus be summarized by the spatiotemporal law of tumor metabolic evolution: during tumor initiation and the hypoxic compensatory phase, the downregulation of ATP5A1 represents a survival compromise to trigger the Warburg effect and oncogenic signaling (e.g., Wnt, ROS). However, during malignant progression and distant metastasis, the reactivation or overexpression of ATP5A1 becomes a critical factor to fulfill extreme energy demands and sustain the high metabolic burden of the tumor.

### 3.2 Functional roles and mechanisms of ATP5A1 in non-neoplastic diseases

The functional roles and mechanisms of ATP5A1 in non-neoplastic diseases are summarized in Table 2.

**TABLE 2:** Expression patterns and mechanisms of ATP5A1 in non-neoplastic diseases.

System	Specific disease/Physiological state	Expression or state change	Core mechanisms and pathological effects	References
Immune system	Myeloid cell activation	Upregulated/activated	Provides energy support for phagocytosis and superoxide anion production	Cao et al. (2020)
	Severe infection/systemic inflammation	Dysregulated expression	Exacerbates hyperinflammation and disrupts antibody secretion networks	Qi et al. (2021)
	Rheumatoid arthritis	Genetic polymorphism	Affects efficacy and toxicity of immunosuppressants such as leflunomide	Soukup et al. (2015)
Neuropsychiatric system	Alzheimer’s disease	Early downregulation	Induces synaptic energy depletion; recombinant ATP5A1 binds LPS to inhibit neuroinflammation	Liang et al. (2008), Yue et al. (2021)
	Parkinson’s disease	Functional interaction	Otx2 translocates to mitochondria and interacts with ATP5A1, enhancing catalytic efficiency and rescuing neurotoxicity	Xue et al. (2022)
	Schizophrenia	Systemic downregulation	OXPHOS deficiency leads to neuronal metabolic crisis and disrupted brain circuitry	Katz Shroitman et al. (2023)
Cardiovascular system	Ischemia–reperfusion injury	Pathological cleavage	Calpain-1 disrupts ATP5A1 structural framework, triggering mPTP opening, ROS burst, and cardiomyocyte death	Zheng et al. (2021)
	Septic cardiomyopathy	Dysfunction/cleavage	SIRT3 impairment shuts down ATP production; NMN supplementation restores SIRT3 and rescues cardiac function	Koentges et al. (2019), Cao et al. (2023)
	Hypertensive cardiac hypertrophy	Early upregulation → late downregulation	Early compensation meets workload demands; late decompensation leads to energy depletion and heart failure	Liu et al. (2016), Meng et al. (2009), Byun et al. (2019)
Metabolic system	Hepatic steatosis	Impaired activity	*Helicobacter pylori* virulence factors bind ATP5A1, activating mTORC1–SREBP1 axis and driving lipid accumulation	Jin et al., (2025)
	Skeletal muscle fat infiltration	Ubiquitin-mediated degradation	GADD45 A recruits TRIM25 to accelerate ATP5A1 degradation; EGCG rescues expression	You et al. (2023)
	Osteoporosis	Suppressed expression	Dexamethasone induces mitochondrial energy deprivation, blocking osteogenic differentiation of bone marrow mesenchymal stem cells	Hong et al. (2011)
	Gestational diabetes	Aberrant promoter methylation	Transmitted as epigenetic imprint to offspring, increasing long-term metabolic syndrome risk	Haertle et al. (2017)
Reproductive system	Asthenozoospermia	Downregulated (seminal exosomes)	Bioenergetic defect in sperm midpiece impairs motility; potential non-invasive diagnostic biomarker	Panner Selvam et al. (2019)
	Oocyte development	Differential enrichment	Reflects mitochondrial activity; key reference for assessing oocyte maturation and developmental potential	Torner et al. (2008)
	Environmental toxicity exposure	Disrupted mitochondrial energy network	Bisphenols/pesticides induce mitophagy and apoptosis in cumulus-oocyte complex, leading to loss of developmental competence	Ma C. et al., (2024)

#### 3.2.1 Immune-mediated diseases

In immune-mediated diseases and severe infection-associated inflammatory responses, ATP5A1-mediated reprogramming of mitochondrial energy metabolism serves as a key metabolic node involved in regulating immune cell activation, polarization, and the intensity of immune responses. Within the innate immune system, the bactericidal and clearance functions of macrophages and neutrophils are highly dependent on mitochondrial metabolic support (West et al., 2011). Studies have demonstrated that overexpression of angiotensin-converting enzyme (ACE) significantly upregulates core electron transport chain proteins, including ATP5A1, through its catalytic activity, thereby substantially increasing intracellular ATP production and the levels of tricarboxylic acid cycle intermediates. This provides essential energy and metabolic substrates for superoxide generation and efficient phagocytosis in myeloid cells (Cao et al., 2020). However, in the context of severe infection-induced systemic inflammatory responses, dysregulation of this metabolic pathway becomes a pathological driver. Aberrant expression of OXPHOS-related genes, including *ATP5A1*, in patient-derived monocytes and plasma cells not only exacerbates pathogenic hyperinflammation but also disrupts normal antibody secretion networks (Qi et al., 2021). Furthermore, genetic variants of *ATP5A1* have important clinical translational relevance in the pharmacokinetics of classical autoimmune diseases, such as rheumatoid arthritis (RA). SNPs in ATP5A1 are closely associated with the therapeutic efficacy and adverse effect profiles of immunosuppressive agents, including leflunomide (Soukup et al., 2015). These findings overlap in their assertion that ATP5A1 functions not only as a key regulator of immunometabolism but also as a promising pharmacogenomic biomarker, offering a framework for individualized treatment of immune-mediated diseases.

#### 3.2.2 Neuropsychiatric disorders

In the pathological progression of neurodegenerative and psychiatric disorders, ATP5A1-mediated mitochondrial bioenergetic crisis and neuroinflammatory network imbalance constitute a central molecular hub driving neuronal functional decline and aberrant network connectivity. In Alzheimer’s disease (AD), ATP5A1 expression is significantly downregulated in key brain regions during early disease stages, such as the posterior cingulate cortex, leading to energy depletion that is required to maintain synaptic function (Liang et al., 2008). Furthermore, recombinant ATP5A1 has been shown to directly bind lipopolysaccharide (LPS) in the extracellular microenvironment, thereby potently inhibiting excessive activation of microglia and astrocytes and reducing IL-1β accumulation and pathological Tau phosphorylation, thereby exhibiting marked anti-neuroinflammatory potential (Yue et al., 2021).

In Parkinson’s disease (PD), the survival of dopaminergic neurons in the substantia nigra is critically dependent on mitochondrial homeostasis (Scorziello et al., 2020). Recent intervention studies have shown that the exogenous homeodomain transcription factor Otx2 can translocate to mitochondria and physically interact with ATP5A1. This interaction directly enhances the catalytic efficiency of F_1_F_0_-ATP synthase, effectively rescuing MPP^+^-induced neurotoxicity and confirming the decisive role of ATP5A1 in maintaining dopaminergic neuron survival (Xue et al., 2022). Furthermore, in the context of pathological remodeling within higher-order cognitive networks, large-scale transcriptomic meta-analyses of post-mortem brain tissues from patients with schizophrenia have revealed a systematic downregulation of ATP5A1 expression across sexes. This suggests that this potential oxidative phosphorylation defect may be highly associated with a severe neuronal metabolic crisis, which in turn may serve as one of the pathophysiological bases for disrupting normal brain circuit function and triggering cognitive and behavioral abnormalities in schizophrenia (Katz Shroitman et al., 2023).

#### 3.2.3 Cardiovascular diseases

In the cardiovascular system, given the sustained and extreme energy demands of the myocardium, ATP5A1 functions not only as a central component of mitochondrial OXPHOS but also as a key molecular determinant of cardiomyocyte survival and remodeling under various pathological stresses. In acute myocardial infarction and subsequent ischemia–reperfusion injury, severe calcium overload and oxidative stress aberrantly activate the mitochondrial protease calpain-1, leading to pathological cleavage of ATP5A1. This disruption of the rigid F_1_F_0_-ATP synthase framework strongly promotes irreversible opening of the mPTP and a lethal ROS burst, ultimately driving cardiomyocyte necrosis, apoptosis, and collapse of the microvascular microenvironment (Ni et al., 2016; Zheng et al., 2021). In contrast to this structural disruption, septic cardiomyopathy is driven by a systemic inflammatory storm that induces a severe myocardial energy crisis. On the one hand, inflammatory signaling suppresses the expression of upstream regulators of mitochondrial biogenesis, such as PGC-1α. More critically, severe impairment of SIRT3 deacetylase activity leads to functional dysfunction and pathological cleavage of core OXPHOS components, including ATP5A1, thereby effectively shutting down cardiac energy production (Koentges et al., 2019). Recent intervention studies have demonstrated that targeted supplementation with nicotinamide mononucleotide (NMN) to restore intracellular NAD^+^ levels can robustly restore SIRT3 activity. This approach not only prevents aberrant modification and damage of mitochondrial proteins, including ATP5A1, but also significantly reverses sepsis-induced cardiac dysfunction and multiple organ failure (Cao et al., 2023).

In the early stage of compensatory cardiac hypertrophy induced by hypertension, cardiomyocytes exhibit transient metabolic adaptation by upregulating the expression and protein synthesis of metabolic components, including ATP5A1, to meet increased biomechanical workload demands (Liu et al., 2016; Meng et al., 2009). However, with prolonged pressure overload, this metabolic adaptability becomes exhausted, leading to significant downregulation of core OXPHOS genes, including *ATP5A1*. This underlying bioenergetic decompensation and mitochondrial energy depletion ultimately drive the transition from compensatory hypertrophy to decompensated heart failure and irreversible interstitial fibrotic remodeling (Byun et al., 2019). Collectively, these context-dependent mechanisms establish ATP5A1 as a central integrator of ischemic, inflammatory, and biomechanical stress networks in the cardiovascular system.

#### 3.2.4 Metabolic diseases

In the pathological progression of metabolic diseases and systemic endocrine disorders, ATP5A1-mediated mitochondrial bioenergetic imbalance serves as a central molecular hub that drives lipid accumulation, target-organ steatosis, and microvascular complications. In non-alcoholic fatty liver disease (NAFLD) and hepatic steatosis, mitochondrial structural damage and bioenergetic failure serve as major drivers of pathological lipid accumulation. Recent studies have revealed that specific pathogenic factors, such as *Helicobacter pylori* virulence factors, can directly translocate to mitochondria and specifically bind ATP5A1, thereby severely impairing ATP synthase catalytic activity. This physical disruption of the ATP5A1 core component not only weakens hepatic lipid metabolic capacity but also induces excessive mitochondrial ROS production, thereby strongly promoting *de novo* lipogenesis and lipid accumulation in hepatocytes via activation of the mTORC1–SREBP1 signaling axis (Jin et al., 2025). Furthermore, epigenetic regulation has been shown to exert a fundamental protective role against high-fat diet–induced severe hepatic steatosis by maintaining the expression of core metabolic genes, including ATP5A1 (Podrini et al., 2015).

In skeletal muscle metabolic decline and obesity-associated intramuscular lipid infiltration, loss of ATP5A1 expression likewise represents a central pathogenic event. The stress-inducible protein GADD45 A recruits the E3 ubiquitin ligase TRIM25 to accelerate ubiquitin-mediated degradation of ATP5A1, leading to a marked reduction in mitochondrial ATP synthesis and exacerbation of lipid infiltration. Nutritional interventions, such as supplementation with epigallocatechin gallate (EGCG), can effectively downregulate GADD45 A and restore ATP5A1 expression, thereby significantly alleviating skeletal muscle lipid infiltration and metabolic dysfunction (You et al., 2023). Meanwhile, in response to microvascular damage induced by systemic metabolic stressors such as hyperglycemia and hyperlipidemia, activation of specific metabolic regulatory networks, such as the CTRP9–AdipoR1/SIRT1 axis, significantly upregulates the protein expression of NRF1, TFAM, and ATP5A1, thereby restoring mitochondrial biogenesis in affected target organs and providing a defined molecular pathway for intervention in metabolic microvascular complications (Cheng et al., 2016).

Furthermore, severe metabolic dysregulation profoundly affects skeletal remodeling. In glucocorticoid-induced osteoporosis, high-dose dexamethasone not only directly suppresses the expression of ATP synthase family genes but also activates the aberrant CAST–CAPN1–ATP5A1 signaling axis, causing lethal mitochondrial energy deprivation in bone marrow mesenchymal stem cells and completely blocking their osteogenic differentiation potential (Hong et al., 2011).

More profoundly, maternal metabolic stress induced by gestational diabetes mellitus (GDM) can exert cross-generational effects through epigenetic pathways. Within the adverse intrauterine hyperglycemic and hyperlipidemic microenvironment, excessive oxidative stress disrupts the activity of DNA methyltransferases (DNMTs) during early embryonic development. Preliminary clinical cord blood epigenomic studies have confirmed that cord blood samples from the offspring of GDM mothers exhibit specific aberrant DNA methylation patterns in the promoter region of the ATP5A1 gene (Haertle et al., 2017). This epigenetic imprint, targeting a core component of mitochondrial bioenergetics, is preserved long-term as a “metabolic memory” during offspring organogenesis and postnatal development, leading to persistent impairment of baseline oxidative phosphorylation (OXPHOS) function in target organs. Integrating recent mechanistic frameworks regarding the cross-generational epigenetic regulation of obesity and diabetes (Ibrahim, 2022), we propose that the sustained epigenetic reprogramming of key mitochondrial genes, such as ATP5A1, not only compromises metabolic flexibility in response to energy fluctuations but may also serve as a core molecular mechanism mediating the long-term development of insulin resistance, lipid accumulation, and metabolic syndrome in offspring. This transmission from “maternal metabolic decompensation” to “offspring genomic remodeling” further highlights the global regulatory role of ATP5A1 in systemic endocrine responses.

#### 3.2.5 Reproductive system disorders

In reproductive system disorders and abnormal gametogenesis, given the exceptionally high energy demands of sperm motility and oocyte maturation, ATP5A1-mediated mitochondrial OXPHOS homeostasis serves as a central metabolic gatekeeper determining gamete quality and fertilization potential (Almeida-Reis et al., 2024; Panner Selvam et al., 2019). In male infertility, particularly asthenozoospermia, sustained sperm motility is highly dependent on ATP generated by mitochondria assembled in the midpiece of the flagellum. Clinical proteomic studies have revealed that ATP5A1 expression is significantly downregulated in seminal exosomes from affected patients. This bioenergetic defect caused by ATP5A1 deficiency not only directly impairs sperm motility but also highlights its clinical diagnostic value as a non-invasive molecular biomarker for male reproductive dysfunction (Panner Selvam et al., 2019). Similarly, the resumption of meiosis in oocytes and the development of early cloned embryos require substantial mitochondrial energy support. Transcriptomic analyses have demonstrated that, during the selection of oocytes with high developmental potential using brilliant cresyl blue staining, differential enrichment of core electron transport chain genes, including ATP5A1, directly reflects underlying mitochondrial activity and serves as a key molecular indicator for evaluating oocyte *in vitro* maturation and epigenetic reprogramming capacity (Torner et al., 2008). Furthermore, the reproductive microenvironment is highly susceptible to toxic stress from environmental compounds, including bisphenols and fungicidal pesticides. Recent reproductive toxicology studies have demonstrated that these exogenous stressors can directly infiltrate the follicular microenvironment and disrupt the mitochondrial energy metabolism network, mediated by core components such as ATP5A1, thereby inducing severe mitochondrial dysfunction. This mitochondrial dysfunction directly triggers lethal mitophagy and apoptosis in the cumulus–oocyte complex, leading to complete loss of oocyte developmental competence and significantly reducing fertilization rates and high-quality embryo yield in assisted reproductive technologies (Gorczyca et al., 2022; Ma C. et al., 2024). Collectively, these mechanisms establish ATP5A1 not only as an energetic cornerstone that maintains gametogenesis and embryonic development but also as a precise mitochondrial target for clinical intervention in metabolic infertility and for optimizing *in vitro* fertilization systems.

## 4 Clinical translational prospects of ATP5A1

ATP5A1 has long been regarded as a constitutive housekeeping gene responsible for maintaining basal cellular metabolism (Kagawa and Ohta, 1990; Shaw et al., 2011). However, recent advances in translational medicine are fundamentally redefining this paradigm. As a highly dynamic “metabolism–cell fate gating hub,” ATP5A1 is rapidly establishing a continuum spanning early quantitative diagnosis to molecular targeted intervention across multiple disease systems.

### 4.1 Diagnostic applications of ATP5A1

In clinical diagnostics, ATP5A1 and its associated complexes, owing to their high tissue specificity and dynamic expression patterns, have emerged as core biomarkers for evaluating disease onset and progression.

#### 4.1.1 Diagnostic applications in oncology

Histological biomarkers and prognostic stratification: In clear cell renal cell carcinoma (ccRCC), large-scale clinical cohort studies have demonstrated that low ATP5A1 expression is an independent and robust biomarker associated with significantly reduced overall survival (Yuan et al., 2018).

Non-invasive liquid biopsy: For gastrointestinal malignancies characterized by strong dependence on metabolic reprogramming, a peripheral blood-based liquid biopsy model incorporating core homologous subunits of the ATP5A1-containing complex (such as ATP5B and ATP5O) has been developed. This four-protein plasma signature achieved area under the curve (AUC) values of 0.996 in the training cohort and 0.886 in the validation cohort, indicating strong diagnostic performance. In the early non-invasive discrimination of gastric cancer patients from healthy individuals, this model demonstrates superior sensitivity and specificity compared with conventional single-marker carcinoembryonic antigen (CEA) testing, thereby offering substantial clinical utility (Mottaghi-Dastjerdi et al., 2023; Song et al., 2020).

#### 4.1.2 Diagnostic applications in non-neoplastic diseases

Early warning of ischemia in the cardiovascular system: Following acute myocardial infarction, a marked reduction in ATP5A1 expression in circulating biomarkers or myocardial biopsy samples (below physiological baseline levels) serves as a core indicator for precisely assessing local monocyte/macrophage inflammatory infiltration and irreversible cardiac functional collapse (Kuang et al., 2024).

Blood-based early screening in neuropsychiatric disorders: In large-scale diagnostic cohorts of idiopathic PD, a peripheral blood gene expression microarray model comprising 100 probes has been developed. Using the “systemic downregulation” of mitochondrial bioenergetic genes, represented by ATP5A1, in peripheral blood mononuclear cells as a threshold, this model enables large-scale non-invasive screening prior to the onset of severe motor dysfunction (Shamir et al., 2017).

Pathological stratification in reproductive and metabolic diseases: In male asthenozoospermia, ATP5A1 protein abundance in the sperm midpiece and seminal exosomes serves as a standardized diagnostic marker for evaluating sperm progressive motility (Panner Selvam et al., 2019). In the progression of NAFLD toward fibrosis, a characteristic sharp reduction in ATP5A1 protein abundance in biopsy tissues constitutes a key molecular criterion for the precise diagnosis of hepatic metabolic decompensation and irreversible activation of quiescent hepatic stellate cells (i.e., fibrosis) (García-Ruiz et al., 2014; Yin et al., 2020; Niu et al., 2022).

### 4.2 Clinical interventions targeting ATP5A1 and its complexes

Beyond serving as a diagnostic target, highly selective mitochondria-targeted interventions directed at ATP5A1 have progressed from basic pharmacological exploration to advanced-phase clinical trials.

#### 4.2.1 Frontier progress in tumor-targeted interventions

Translational breakthroughs of Gboxin and its biomimetic nano-formulations: The small-molecule agent Gboxin functions as an oxidative phosphorylation inhibitor. Although it demonstrates exceptional specificity for mitochondrial ATP synthase in glioblastoma, its early clinical translation has been hindered by the free molecule’s rapid clearance and poor blood-brain barrier (BBB) penetration rate (typically <1%) (Shi et al., 2019). A major breakthrough currently advancing through late-stage preclinical evaluation is the development of biomimetic nanomedicines comprising Gboxin encapsulated within cancer cell-mitochondria hybrid membranes. By exploiting homotypic targeting effects, this formulation not only achieves a multi-fold increase in drug accumulation within the targeted glioma region but also significantly prolongs median overall survival in orthotopic patient-derived xenograft (PDX) mouse models. Currently, this delivery system is undergoing an in-depth pharmacokinetic and toxicological evaluation prior to the Investigational New Drug (IND) application. It must be emphasized that, although positive results have been achieved in vivo murine models, these therapeutic effects have not yet been confirmed in human clinical trials, and its translational application remains of a strictly preclinical nature (Zou et al., 2023; Liu et al., 2025).

Targeted elimination of chemoresistant clones by Aurovertin B. As a canonical allosteric inhibitor of the F1 catalytic domain of ATP synthase, the current translational focus of Aurovertin B is centered on overcoming chemotherapy resistance (van Raaij et al., 1996). In multiple organoid and animal models of paclitaxel-resistant triple-negative breast cancer (TNBC), Aurovertin B hybrids have been demonstrated to thoroughly dismantle the compensatory oxidative phosphorylation mechanisms upon which resistant cells rely for survival. Its treatment regimen as a “chemotherapy metabolic sensitizer” in combination with classical taxanes is undergoing early targeted dose exploration and safety evaluation. At present, these findings are completely confined to *in vitro* organoids and animal models, and immense systemic toxicity evaluation obstacles still need to be overcome before actual clinical translation (Wu et al., 2020; Ma L. F. et al., 2024).

#### 4.2.2 Clinical application prospects in non-neoplastic diseases

Elamipretide represents a milestone in mitochondria-targeted metabolic intervention (Sabbah et al., 2025). By specifically binding to cardiolipin on the inner mitochondrial membrane of cardiomyocytes, it structurally stabilizes the “ATP synthasome” complex that includes ATP5A1 (Pharaoh et al., 2023; Chavez et al., 2020). In Phase II/III clinical trials for primary mitochondrial cardiomyopathies, such as Barth syndrome (TAZPOWER trial, NCT03098797), elamipretide significantly improved myocardial energy coupling and slowed the decline in left ventricular ejection fraction (Reid Thompson et al., 2021). In September 2025, the U.S. FDA granted accelerated approval to elamipretide, making it the first approved targeted therapy for mitochondrial disorders and marking the transition of cardiolipin- and bioenergetics-centered interventions into routine clinical use (Zhao et al., 2026; Shirley, 2026).

Target-organ metabolic remodeling by GLP-1 receptor agonists:GLP-1 receptor agonists have extended beyond their traditional roles in glycemic control and weight management. In recent large-scale cardiovascular outcome trials and clinical studies of metabolic dysfunction-associated steatohepatitis, agents such as liraglutide and semaglutide have demonstrated robust target-organ–protective effects independent of glucose-lowering. The underlying mechanism involves activation of the AMPK/SIRT1 energy signaling axis in target cells, including hepatic stellate cells and cardiomyocytes (Wang et al., 2018). This activation restores and upregulates key bioenergetic components, including ATP5A1, under pathological conditions, enhances fatty acid oxidation capacity, and suppresses organ lipotoxicity and fibrotic progression (Xu et al., 2014; Yang et al., 2020).

## 5 Non-canonical biological functions of ATP5A1: new frontiers reshaping current paradigms

Traditional cell biology paradigms have strictly compartmentalized ATP5A1 to the mitochondrial matrix and inner membrane system. However, emerging research focusing on spatiotemporal dynamics is fundamentally challenging this conventional understanding. Accumulating evidence demonstrates that ATP5A1 exhibits highly dynamic, non-canonical subcellular localizations; these “moonlighting” roles, extending beyond the classical perspective, are redefining its biological dimensions in the context of complex diseases.

### 5.1 Nuclear translocation and epigenetic transcriptional remodeling

Breakthrough mechanistic studies have revealed that under specific malignancies and extreme microenvironmental stresses, ATP5A1 can escape the physical constraints of its mitochondrial targeting sequence to undergo distinct nuclear translocation. Upon entering the nucleus, ATP5A1 completely divests itself of its traditional metabolic role in catalyzing ATP synthesis, acting instead as a core co-factor of transcriptional regulatory complexes to directly mediate the expression reprogramming of downstream target genes and the alternative splicing networks of cancer-associated genes (Song Ba et al., 2021). This identity transition from a “metabolic motor” to an “epigenetic and transcriptional regulator” allows mitochondrial energetic stress signals to be directly and rapidly communicated to the chromatin level, providing a fresh perspective on cellular adaptive evolution.

### 5.2 Ecto-F1-ATPase and microenvironmental communication

Furthermore, high-resolution subcellular tracking techniques have revealed that ATP5A1-containing complex V structures can undergo dynamic translocation to the plasma membrane via specialized vesicular transport mechanisms, forming extracellularly exposed ecto-F1-ATPase (Chang et al., 2023). Cell surface-localized ATP5A1 directly participates in the regulation of purinergic signaling within the stromal microenvironment and immune synapses by modulating the homeostasis of extracellular ATP and ADP.

The confirmation of these non-canonical roles not only transcends traditional organelle functional boundaries but also provides a compelling avenue for clinical translation: specifically targeting cell-surface ecto-F1-ATPase or blocking its nuclear translocation pathways holds promise for precisely disrupting pathological communication while maximally mitigating the systemic off-target toxicities to healthy tissues typically associated with conventional mitochondrial inhibitors.

## 6 Conclusion

For a long time, ATP5A1 has been viewed solely as a “housekeeping gene” for maintaining basal metabolism and a static component in the oxidative phosphorylation network. However, current evidence has completely reshaped this perception: ATP5A1 is, in fact, a highly dynamic “metabolic-cell fate gating node.” It not only precisely regulates energy homeostasis through post-translational modifications and genetic polymorphisms, but also demonstrates profound potential driving value in tumor metabolic reprogramming, cardiovascular stress injury, neurodegeneration, and immunometabolic polarization. ATP5A1-based peripheral blood biopsies, prognostic biomarkers, and targeted modulators provide promising translational avenues for this field, but currently, most still remain in the preclinical exploration stage. Transitioning from basic associations to definitive causal validation and clinical precision treatment remains a chasm that urgently needs to be bridged in the future.

### 6.1 Current limitations in ATP5A1 research

Despite substantial progress in ATP5A1 research, several critical limitations remain to be addressed for deeper mechanistic understanding and large-scale clinical application:

Experimental validation of the heterogeneity switch mechanism is required.: Although this review proposes a spatiotemporal trade-off hypothesis based on microenvironmental stress and metabolic evolutionary stages to elucidate the paradoxical role of ATP5A1 across different tumors, systematic and direct *in vivo* evidence identifying the core regulatory switch underlying this context-dependent phenomenon remains absent. Future investigations must urgently leverage multi-omics approaches to decipher the precise molecular networks governing this metabolic transition node at single-cell resolution.

Toxicity of broad inhibition and delivery barriers: ATP synthase is essential for the survival of cardiomyocytes and neurons, and conventional inhibitors carry a high risk of severe toxicity (Nafi et al., 2025). Moreover, achieving effective targeted delivery, such as crossing the blood–brain barrier or penetrating dense solid tumor microenvironments, remains a major obstacle to drug development (Sanchez-Aranguren et al., 2025).

Substantial gaps in non-canonical functional exploration: Most existing studies focus on the canonical bioenergetic role of ATP5A1 within the mitochondrial matrix, whereas its potential non-canonical functions remain insufficiently explored. Emerging evidence indicates that ATP5A1 can translocate to the nucleus to directly participate in transcriptional regulation and alternative splicing of target genes (Song Ba et al., 2021) and can also relocalize to the plasma membrane as ecto-F_1_-ATPase to mediate microenvironmental communication (Chang et al., 2023). These structural and non-metabolic functions remain to be systematically elucidated.

Potential bias from neglecting age and sex as key biological variables: Modern precision medicine and translational research highly emphasize integrating age and sex as independent biological variables. In neuropsychiatric disorders, large-scale meta-analyses have confirmed a significant “cross-sex” systemic downregulation of ATP5A1 in the brains of patients with schizophrenia, suggesting that the role of ATP5A1 in the underlying metabolic deficits of this disease possesses universal applicability across both sexes (Katz Shroitman et al., 2023). However, systematic analyses of sex differences and age stratification remain severely lacking in the vast majority of current studies concerning ATP5A1 in malignant tumor progression, ischemic cardiovascular diseases, and immune polarization. Given that mitochondrial oxidative phosphorylation efficiency undergoes a progressive physiological decline during natural aging, and sex hormones (e.g., estrogen) have been proven to profoundly influence cellular energy fluxes and antioxidant stress capacities via targeted mitochondrial receptors (Lejri et al., 2018), the expression dynamics and pathogenic thresholds of ATP5A1 are highly likely to exhibit significant heterogeneity across different age groups and sexes. Failure to control for these critical demographic variables may not only mask the true effects of ATP5A1 in specific subpopulations but also severely limit the generalizability assessment of relevant targeted therapeutics. Therefore, future preclinical model development and large-sample cohort analyses regarding ATP5A1 and its complexes must rigorously incorporate sex and age stratification strategies to comprehensively elucidate how these core variables reshape the ATP5A1-mediated metabolic-signaling networks.

Imbalance in evidence hierarchy and the translational gap. Despite the integration of multidimensional data in current ATP5A1 research, a pronounced imbalance exists regarding evidence weight and clinical translational efficacy. A vast majority of contemporary mechanistic discoveries—particularly those detailing targeted interventions and the precise remodeling of signaling networks—remain disproportionately reliant on singular *in vitro* immortalized cell lines (e.g., gastric cancer MGC803 and lung cancer A549). While advantageous for genetic manipulation, these *in vitro* models are entirely detached from the authentic tissue stroma, immune crosstalk, and vascular microenvironment, thereby failing to faithfully recapitulate the metabolic heterogeneity of primary tumors. Conversely, existing high-level clinical evidence predominantly derives from large-scale retrospective multi-omics databases or tissue microarrays. Although such data hold significant clinical weight in establishing prognostic biomarkers, they typically yield only statistical correlations devoid of causal substantiation. Currently, there is a relative paucity of “bridge models”—such as patient-derived organoids (PDOs), primary cell co-culture systems, or humanized patient-derived xenograft (PDX) mice—capable of connecting these two evidentiary extremes. This disconnect within the evidence hierarchy severely restricts the translational efficiency of ATP5A1-targeted strategies from the laboratory bench to real-world clinical applications. Moving forward, there is an urgent need to conduct cross-validation that seamlessly integrates mechanistic insights with phenotypic outcomes in prospective cohorts with adequate clinical sample sizes.

### 6.2 Future perspectives and breakthroughs in ATP5A1 research

Targeting the aforementioned challenges, future breakthroughs in the ATP5A1 field should focus on the deep integration of multidisciplinary approaches and advanced foundational technologies:

Integration of single-cell and spatial multi-omics: It is imperative to leverage single-cell RNA sequencing and spatial multi-omics to precisely map the dynamic expression landscape of ATP5A1 within complex pathological tissues (such as dense solid tumors or inflammation-driven myocardial fibrotic remodeling regions), thereby comprehensively elucidating tissue heterogeneity and intercellular metabolic symbiotic networks (Zhang et al., 2024; McCallinhart et al., 2025).

Targeted nanodelivery and protein degradation technologies: Pharmacological development should shift from “global inhibition” toward “precision delivery”. This includes the design of nanocarriers responsive to specific pathological microenvironments (e.g., acidic conditions or high ROS levels), as well as the introduction of mitochondria-targeted protein degradation technologies (PROTACs) to selectively eliminate pathological ATP5A1 while minimizing off-target toxicity in the heart and brain (Tong et al., 2024; Yamada et al., 2024).

Combination strategies targeting minimal residual disease: To prevent compensatory metabolic bypass, ATP5A1-targeted interventions should be combined with immune checkpoint inhibitors (e.g., PD-1/PD-L1) or chemotherapy. Such synergistic approaches may induce metabolic crisis, relieve immunosuppression, and facilitate the complete eradication of dormant minimal residual clones (Boreel et al., 2021).

Routine implementation of pharmacogenomics for individualized therapy: Incorporating ATP5A1 SNP profiling into routine pre-treatment screening for systemic diseases may enable metabolism-guided individualized dosing of immunosuppressants (such as leflunomide) and improve toxicity prevention, thereby establishing safer precision medicine strategies (Soukup et al., 2015).

In summary, ATP5A1 is not only a key regulator of fundamental cellular bioenergetics but also a critical component in addressing complex multisystem diseases. With continued advances in targeted technologies and progressive refinement of its molecular regulatory network, ATP5A1-centered diagnostic and therapeutic strategies are poised to demonstrate transformative clinical value in the era of precision medicine.
